# Synthesis, Crystal Structure and Thermal Decomposition of the New Cadmium Selenite Chloride, Cd_4_(SeO_3_)_2_OCl_2_

**DOI:** 10.1371/journal.pone.0097175

**Published:** 2014-05-20

**Authors:** Faiz Rabbani, Humayun Ajaz, Iwan Zimmermann, Mats Johnsson

**Affiliations:** 1 Department of Chemistry, COMSATS Institute of Information Technology, Abbottabad, Khyber Pakhtunkhwa, Pakistan; 2 Department of Chemistry, University of Engineering and Technology, Lahore, Pakistan; 3 Department of Materials and Environmental Chemistry, Stockholm University, Stockholm, Sweden; Martin-Luther-Universität Halle-Wittenberg, Germany

## Abstract

A synthetic study in the Cd-Se-O-Cl system led to formation of the new oxochloride compound Cd_4_(SeO_3_)_2_OCl_2_ via solid state reactions. The compound crystallizes in the orthorhombic space group *Fmmm* with cell parameters *a* = 7.3610(3) Å, *b* = 15.4936(2) Å, *c* = 17.5603(3) Å, Z = 8, S = 0.969, F(000) = 2800, R = 0.0185, R_w_ = 0.0384. Single crystal X-ray data were collected at 293 K. The crystal structure can be considered as layered and the building units are distorted [Cd(1)O_6_] octahedra, distorted [Cd(2)O_8_] cubes, irregular [Cd(3)O_4_Cl_2_] polyhedra and SeO_3_E trigonal pyramids. There are two crystallographically unique Cl atoms that both are half occupied. Thermogravimetric studies show that the compound starts to decompose at 500°C. The crystal structure of the new compound is closely related to the previously described compound Cd_4_(SeO_3_)_2_Cl_4_(H_2_O).

## Introduction

Several oxochloride compounds have previously been synthesized containing Se^4+^ that has a stereochemically active lone pair; *e.g.* Co_5_(SeO_3_)_4_Cl_2_ [1], Zn_2_SeO_3_Cl_2_ [2], Cu_5_(SeO_3_)_2_O_2_Cl_2_ [3], Cu_9_Se_4_O_14_Cl_6_ [4], Cu_3_(SeO_2_)_3_Cl_2_ [5], Mo_2_Se_4_O_2_Cl_8_ [6]. In such compounds Se^4+^ almost always only form bonds to oxygen due to its small radius and high Lewis acidity strength, while late transition metals bond to both oxygen and chlorine anions due to their weaker Lewis acidity strength. The stereochemically active lone-pair on Se^4+^ and the chloride ions both act as terminating groups and help to open up the crystal structures leading to an increased probability to form compounds that may show *e.g.* magnetic frustration when the compounds contain magnetic cations because of the increased probability for those ions to take low-dimensional arrangements; examples of such compounds are Cu_2_Te_2_O_5_X_2_ and FeTe_2_O_5_X (X = Cl, Br) [7]–[8]. Irregular coordination around p-element lone-pair cations also lead to an increased possibility to form non-centrosymmetric crystal structures that may show nonlinear optical second harmonic generation (SHG); examples of such compounds are BaNbO(IO_3_)_5_ and BiO(IO_3_) [9]–[10].

The objective of this synthetic work was to search for new oxochloride compounds in the Cd^2+^-Se^4+^-O-Cl system. There are previously only two oxohalides described containing Cd^2+^ and Se^4+^; Cd_4_(SeO_3_)_2_Cl_4_(H_2_O) [11] and Cd_10_(SeO_3_)_8_Br_4_•HBr•H_2_O [12]. The study resulted in finding the compound Cd_4_(SeO_3_)_2_OCl_2_ that turned out to have a crystal structure very similar to the previously described compound Cd_4_(SeO_3_)_2_Cl_4_(H_2_O) [11].

## Experimental

The following chemicals were used as starting materials: CdCl_2_ (BDH Chemicals, 99%) and SeO_2_ (Alfa Aesar, 99.4%). Single crystals of Cd_4_(SeO_3_)_2_OCl_2_ were obtained from a mixture of CdCl_2_:SeO_2_ in the non-stoichiometric molar ratio 1∶2. The mixture was heated at 450°C for 96 h in an evacuated glass tube in a muffle furnace which was further cooled to room temperature at a rate of 10°C/h. The right phase was not formed at reaction temperatures lower than 400°C and reaction temperatures higher than 450°C resulted in lower yield. The synthesis product consists of white Cd_4_(SeO_3_)_2_OCl_2_ single crystals in a matrix of white undetermined powder that most likely was SeO_2_. The new oxochloride was characterized in a scanning electron microscope (S3700N, HITACHI, Japan) equipped with an energy dispersive spectrometer (HORIBA, Japan) confirming the presence and stoichiometry of the elements Cd, Se, Cl and O. Thermogravimetric measurements were carried out in air using a simultaneous TGA/DSC apparatus SDT Q600 from TA instruments. The residue was further characterized using Fourier Transform Infra-Red (FTIR) spectrophotometer {FTIR-4100, JASCO-CE, PerkinElmer, UK}.

Single crystal X-ray diffraction experiments were carried out on an Oxford Diffraction Xcalibur3 diffractometer equipped with a graphite monochromator. The data collection was performed at 293 K using MoK_α_ radiation, λ = 0.71073 Å. Absorption correction and data reduction were performed with the software CrysAlis RED that also was employed for the analytical absorption correction [13]. The structure solution was carried out with *SHELXS97* and the refinement with *SHELXL97* [14] in the WINGX [15] environment. All atoms were refined anisotropically. Crystal data are reported in Table 1. The structural drawings are made with the program DIAMOND [16].

**Table 1:** Crystal data and structure refinement for Cd_4_(SeO_3_)_2_OCl_2_.

Emperical formula	Cd_4_Se_2_O_2_Cl_2_
Formula weight (amu)	790.42
Temperature (K)	293 (2)
Wavelength (Å)	0.7107
Crystal system	Orthorhombic
Space group	Fmmm
Unit cell dimensions	7.3610(3)Å
	15.4936(2)Å
	17.5603(3)Å
Volume	2002.72 (9) Å^3^
Z	8
Density (calculated) (g cm^−3^)	5.243
Absorption coefficient (mm^−1^)	16.187
F(000)	2800
Crystal Size(mm^3^)	0.04×0.04×0.04
θ range for data collection	3.28–32.06
Index ranges	−10/*h*/8, 22/*k*/22, 25/*l*/25
Reflection collected	4783
Independent reflections	958 (R_int_ = 0.028)
Absorption correction	analytical
Refinement method	Full-matrix least-squares on F^2^
Data/restraints/parameters	958/0/52
Goodness-of-fit of F^2^	0.969
Final *R* indices [*I*>2δ(*I*)]	0.0185
R_1_ index (all data)	R_1_ = 0.0243
	*w*R_2_ = 0.0384
Largest diff. peak and hole(e Å^−3^)	0.825 and −1.274

## Result and Discussion

**Figure 1:**
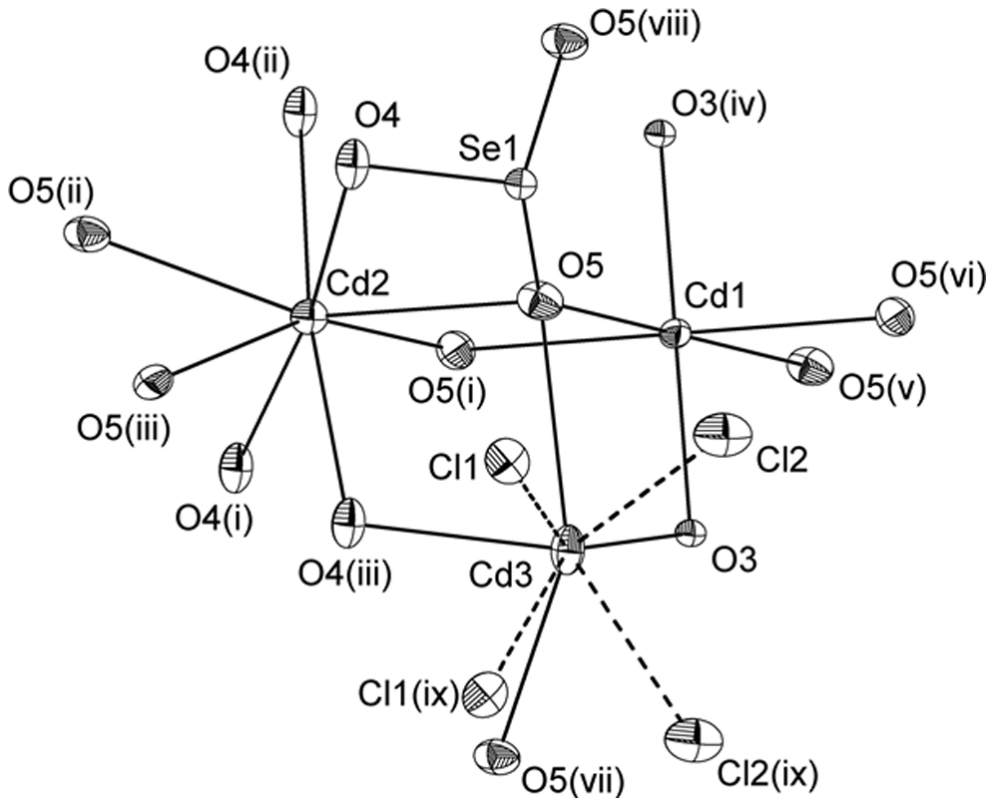
A displacement ellipsoid diagram for Cd_4_(SeO_3_)_2_OCl_2_ showing the coordination around the cations. Atoms Cl(1) and Cl(2) are half occupied and the Cd(3)-Cl bonds are marked as dotted lines. Atomic displacement parameters are given at the 50% probability level. [Symmetry codes: (i) 0.5–x, y, 0.5–z; (ii) x, 0.5–y, 0.5–z; (iii) 0.5–x, 0.5–y, z; (iv) −0.5+x, −y, 0.5–z; (v) 0.5–x, −y, 0.5–z; (vi) x, −y, z; (vii) 1–x, y, z; (viii) −x, y, z; (ix) 1–x, y, −z;].

The new compound Cd_4_(SeO_3_)_2_OCl_2_ crystallizes in the orthorhombic system, space group *Fmmm*. A displacement ellipsoid diagram showing the coordination around the cations is given in Figure 1. EDS-data based on analyses of five different crystals determines the amount of the heavy atoms to be 26.1±0.9 at% Cd, 13.2±0.6at% Se and 13.0±0.5 at% Cl that is in reasonable agreement with the results from the refinement of single-crystal X-ray data; 26.6 at% Cd, 13.3 at% Se and 13.3 at% Cl.

**Figure 2:**
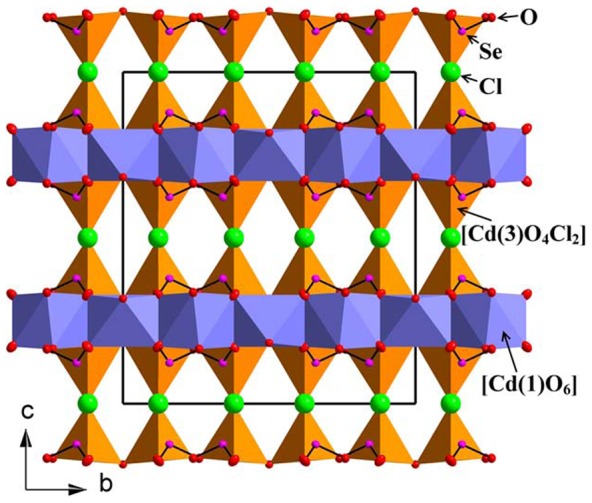
Cd_4_(SeO_3_)_2_OCl_2_ along [100]. The Cl atoms are replaced with a dummy atom inserted halfway in between the half occupied Cl(1) and Cl(2) to replace those two atomic positions in the polyhedra.

**Figure 3:**
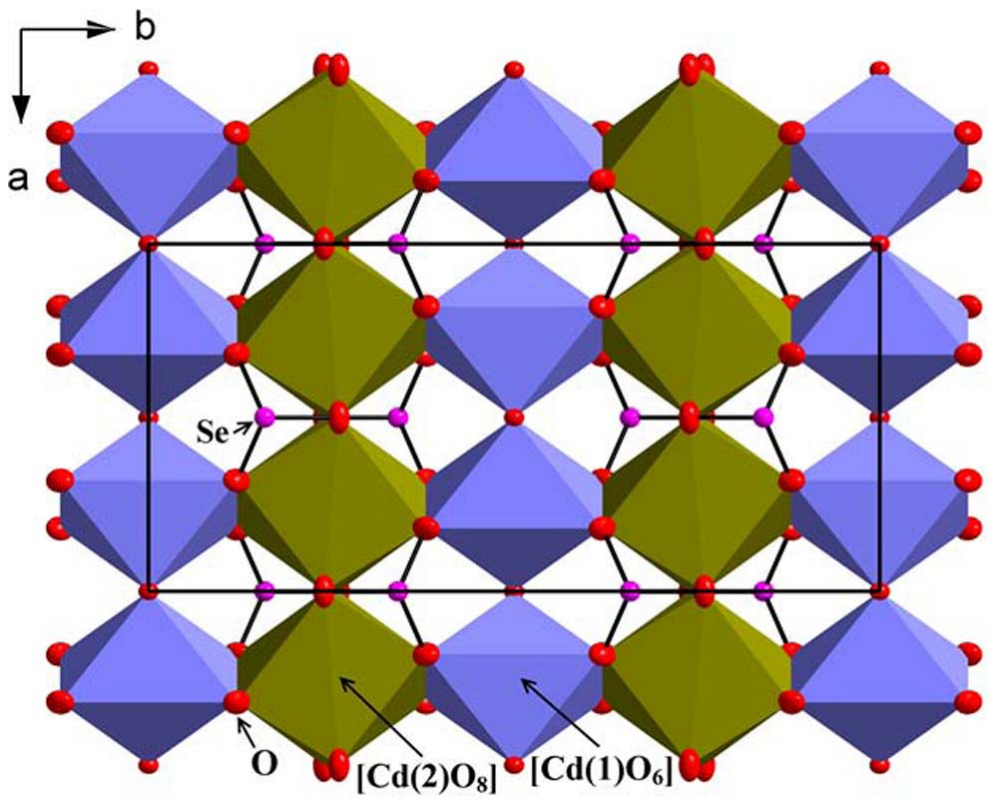
Oxide layer made up of Cd(1)O_6_ and Cd(2)O_8_ polyhedra and SeO_3_ groups, view along [001].

All the cadmium polyhedra are connected to each other to form a 3D network. An overview of the crystal structure of Cd_4_(SeO_3_)_2_OCl_2_ along [100] is shown in Figure 2. There are three unique Cd^2+^ ions having different coordination polyhedra; [Cd(1)O_6_], [Cd(2)O_8_] and [Cd(3)O_4_Cl_2_]. The distorted octahedron [Cd(1)O_6_] has Cd – O distances in the range 2.182(2) –2.379(2) Å and the distorted cube [Cd(2)O_8_] has bond distances in the range 2.379(7)–2.476(2) Å. Those two Cd-polyhedra are connected via corner- and edge sharing to form oxide layers in the crystal structure, see Figure 3. The Cd(3)-atom coordinate four oxygen and four half occupied Cl-positions, two Cl(1) and two Cl(2), that altogether form a square antiprism, see Figure 1. The half occupied Cl(1) atoms show a very short Cl(1)-Cl(1) distance that is 2.179(4) Å and also a very short Cl(2)-Cl(2) distance that is 1.496(5) Å. For the structural drawings the half occupied Cl(1) and Cl(2) atoms are treated as one fully occupied Cl-atom positioned in between Cl(1) and Cl(2) to yield an irregular [Cd(3)O_4_Cl_2_] polyhedron with Cd-O bond lengths in the range 2.146(2)–2.614(2) Å and Cd-Cl distances in the range 2.591(2)–2.698(2).

There is only one crystallographically unique Se atom having the classic trigonal pyramidal [SeO_3_] coordination. The Se-O distances are in the range 1.690(2)–1.733(3) Å. Those polyhedra are isolated and do not polymerize. This is in accordance with earlier observations that SeO_3_ only seldom link to other Se-coordination polyhedra [17].

The [Cd(1)O_6_] polyhedra share corners with two other polyhedra of the same kind to form chains along [100]. Those chains are further connected by edge sharing to chains of edge sharing [Cd(2)O_8_] polyhedra to form oxide layers parallel to (001), see Figure 3. These layers are further linked to the [Cd(3)O_4_Cl_2_] polyhedra via edge sharing. The [Cd(3)O_4_Cl_2_] connect to each other via the Cl-atoms to build the 3D structure, see Figure 2. The [SeO_3_] polyhedra are positioned so that the lone-pairs point out from the oxide layers. Each [SeO_3_] group share corners with two [Cd(1)O_6_] and three [Cd(3)O_4_Cl_2_] and via edge sharing to two [Cd(2)O_8_] polyhedra.

Bond-valence sum calculations yield the following reasonable values; 2.05 for Cd(1), 1.96 for Cd(2), 1.90 for Cd(3), 4.01 for Se(1), 1.98 for O(3), 2.17 for O(4), 2.02 for O(5). Calculations are not made for the Cl-ions due to their half occupancy. The calculations are carried out according to Brown and the r_0_ values used were 1.811 for Se-O and 1.904 for Cd-O and with *B* = 0.37 [18].

The crystal structure of Cd_4_(SeO_3_)_2_OCl_2_ is very similar to the previously described compound Cd_4_(SeO_3_)_2_Cl_4_(H_2_O) [11]. The two structures are considered to be isostructural and a main difference is that the half occupied Cl-positions in Cd_4_(SeO_3_)_2_OCl_2_ are fully occupied in Cd_4_(SeO_3_)_2_Cl_4_(H_2_O), it is, however, not clear from the structure description of the latter compound where the water molecules are located.

TGA analysis in air show that Cd_4_(SeO_3_)_2_OCl_2_ is stable up to 500°C. There is an initial weight loss due to evaporation of adsorbed water (≈1.5%) up to this temperature. The decomposition of the compound takes place in one main step in the temperature range 500–750°C which corresponds to release of two mole SeO_2_ and one mole CdCl_2_ to yield amorphous CdO as the remaining solid product. Powder X-ray diffraction show that the residuals after thermal decomposition is amorphous and IR-spectra show that there are Co-O vibrations, see Supporting Information, Figure S3. The observed weight loss ≈52.5% is close to the calculated (51.3%) for the product CdO. The decomposition curve is available in Supporting Information, Figure S1.

## Conclusions

A new compound Cd_4_(SeO_3_)_2_OCl_2_ was obtained via solid state reactions. It crystallizes in the orthorhombic space group *Fmmm* with the unit cell *a* = 7.3610(3) Å, *b* = 15.4936(2) Å, *c* = 17.5603(3) Å and Z = 8. The building units in the crystal structure are [Cd(1)O_6_], [Cd(2)O_8_], [Cd(3)O_6_Cl_2_] and [SeO_3_] polyhedra. The Cd-polyhedra are linked by corner and edge sharing to build the 3D framework. The crystal structure can be regarded as layered where the oxide layers are connected via Cl-bridges. Thermal gravimetric analysis show that the compound is stable up to 500°C where it starts to decompose.

## Supplementary Material

Crystallographic information files has been sent to Fachinformationzentrum Karlsruhe, Abt. PROKA, 76344 Eggenstein-Leopoldshafen, Germany (fax +49-7247-808-666; email: crysdata@fiz-karlsruhe.de), and can be obtained on quoting the deposit number CSD-426650. Atomic coordinates, selected atomic distances, angles, a graph showing the thermogravimetric decomposition and analysis of the decomposition product are shown in the Supporting Information; Table S1–S3, Figure S1–S3.

## Supporting Information

Figure S1 TG and DTA analysis of Cd_4_(SeO_3_)_2_OCl_2_ using a SDT-Q600 (TA Instrument) in air. The samples of approximately 15 mg were heated in an alumina crucible from room temperature to 800°C at a rate of 5°C/min.
(PDF)

Figure S2 Powder X-ray diffractogram of the residuals after thermal decomposition at 800°C in the TG. No diffraction peaks can be observed due to that the sample is amorphous after thermal decomposition.
(PDF)

Figure S3 Infrared Spectrum of sample residue after TGA. The sample was run on TGA instrument up to 800°C and residue was further characterized using Fourier Transform Infra-Red (FTIR) spectrophotometer {FTIR-4100, JASCO-CE, PerkinElmer, UK} to study the vibrational analysis.
(PDF)

Table S1 Fractional atomic coordinates and isotropic or equivalent isotropic displacement parameters (Å^2^).
(PDF)

Table S2 Selected bond distances and angles (Å,^o^) in Cd_4_(SeO_3_)_2_OCl_2_.
(PDF)

Table S3 Results from Bond Valence Sum (BVS) calculations for Cd_4_(SeO_3_)_2_OCl_2_.
(PDF)

## References

[1] Becker R, Prester M, Berger H, Lin P, Johnsson M, et al (2007) Crystal structure and magnetic properties of two new cobalt selenite halides: Co5(SeO_3_)4X2 (X = Cl, Br). J Solid State Chem 180: 1051–1059.

[2] Johnsson M, Törnroos KW (2007) Zinc selenium oxochloride, β-Zn2(SeO3)Cl2, a synthetic polymorph of the mineral sophiite. Acta Cryst C63: i34–i36. 10.1107/S0108270107012541 17478893

[3] Galy J, Bonnet JJ, Andersson S (1979) The crystal structure of a new oxide chloride of copper(lI) and selenium(lV) Cu5Se2O8Cl2. Acta Chem Scand A33: 383–389.

[4] Krivovichev SV, Filatov SK, Semenova TF, Rozhdestvenskaya IV (1998) Crystal chemistry of inorganic compounds based on chains of oxocentered tetrahedra I. Crystal structure of chloromenite, Cu9O2(SeO3)4Cl6. Z. Kristallographie 213: 645–649.

[5] Becker R, Berger H, Johnsson M (2007) Monoclinic Cu3(SeO3)2Cl2: an oxohalide with an unusual CuO_4_Cl trigonal-bipyramidal coordination. Acta Cryst C63: i4–i6. 10.1107/S0108270106050621 17206031

[6] Beck J (1995) Synthese, Struktur und Phasenumwandlung von Se4(MoOCl4)2. Z Anorg Allgem Chemie 621: 131–136.

[7] Johnsson M, Törnroos KW, Mila F, Millet P (2000) Tetrahedral Clusters of Copper(II): Crystal Structures and Magnetic Properties of Cu_2_Te_2_O_5_X_2_(X = Cl, Br). Chem Mater 12: 2853–2857.

[8] Becker R, Johnsson M, Kremer RK, Klauss H-H, Lemmens P (2006) Crystal structure and magnetic properties of FeTe_2_O_5_X (X = Cl, Br): A frustrated spin cluster compound with a new Te(IV) coordination polyhedron. J Am Chem Soc 128: 15469–15475. 17132014 10.1021/ja064738d

[9] Sun C-F, Hu C-L, Xu X, Ling J-B, Hu T, et al (2009) BaNbO(IO_3_)_5_: A New Polar Material with a Very Large SHG Response. J Am Chem Soc 131: 9486–9487. 19545152 10.1021/ja9030566

[10] Nguyen SD, Yeon J, Kim S-H, Halasyamani PS (2011) BiO(IO_3_): A New Polar Iodate that Exhibits an Aurivillius-Type (Bi2O2)2+ Layer and a Large SHG Response. J Am Chem Soc 133: 12422–12425. 21776958 10.1021/ja205456b

[11] Chen WT, Wei KT, Miao XF (2011) Hydrothermal Synthesis and Crystal Structure of a Novel Selenite-Chloride: [Cd_4_(SeO_3_)_2_Cl_4_(H_2_O)]_n_ with a Three-dimensional Framework. Chinese J Struct Chem 30: 1798–1802.

[12] Chen WT, Wang MS, Wang GE, Chen HF, Guo GC (2013) Solid-state synthesis, structure and properties of a novel open-framework cadmium selenite bromide: [Cd_10_(SeO_3_)_8_Br_4_]·HBr·H_2_O. J Solid State Sciences 204: 153–158.

[13] Oxford Diffraction (2007) CrysAlis CCD and CrysAlis RED. Oxford Diffraction Ltd., Abingdon, Oxfordshire, England.

[14] Sheldrick GM (2008) A short history of SHELX. Acta Cryst (2008) A64: 112–122. 10.1107/S0108767307043930 18156677

[15] Farrugia LJ (1999) WinGX suite for small-molecule single-crystal crystallography. J Appl Crystallogr 32: 837–838.

[16] Brandenburg K (2001) DIAMOND. Crystal Impact GbR, Bonn, Germany.

[17] Mao JG, Jiang HL, Kong F (2008) Structures and properties of functional metal selenites and tellurites. Inorg Chem 47: 8498–8510. 18821816 10.1021/ic8005629

[18] Brown ID, Altermatt D (1985) Bond-valence parameters obtained from a systematic analysis of the Inorganic Crystal Structure Database. Acta Cryst B41: 244–247.

