# Supplementary material for: Synthesis, Crystal Structure and Thermal Decomposition of the New Cadmium Selenite Chloride, Cd4(SeO3)2OCl2
Source: PLoS One. 2014 May 20;9(5):e97175. doi: 10.1371/journal.pone.0097175 (PMC4028199; doi:10.1371/journal.pone.0097175)
Supplement: Figure S1 — TG and DTA analysis of Cd4(SeO3)2OCl2 using a SDT-Q600 (TA Instrument) in air. The samples of approximately 15 mg were heated in an alumina crucible from room temperature to 800°C at a rate of 5°C/min. (PDF) [file pone.0097175.s001.pdf]

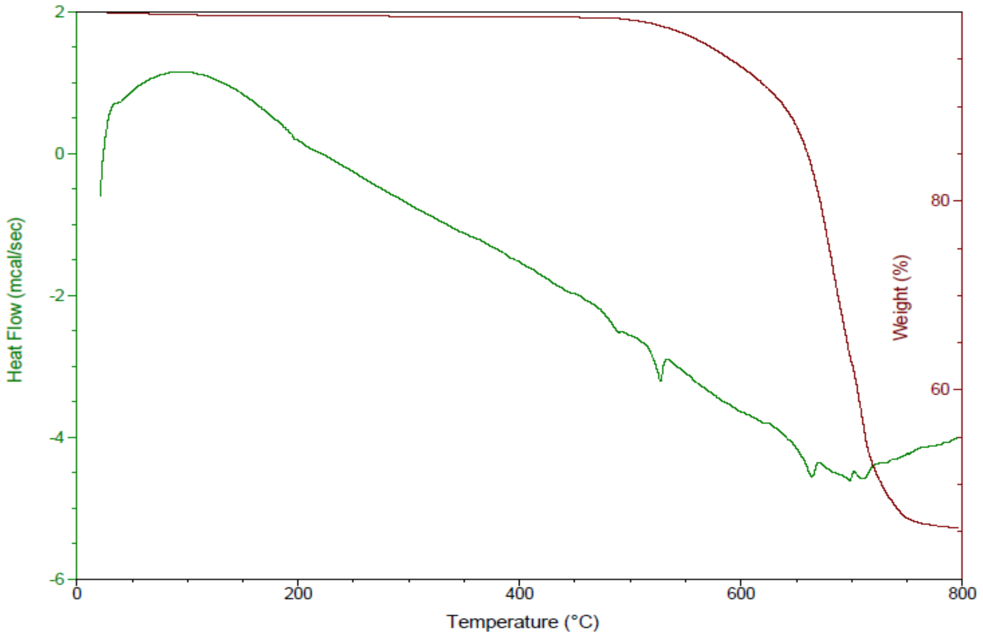

Figure S1 TG and DTA analysis of $Cd_4(SeO_3)_2OCl_2$ using a SDT-Q600 (TA Instrument) in air. The samples of approximately 15mg were heated in an alumina crucible from room temperature to 800°C at a rate of 5°C/min.
