# Supplementary material for: Synthesis, Crystal Structure and Thermal Decomposition of the New Cadmium Selenite Chloride, Cd4(SeO3)2OCl2
Source: PLoS One. 2014 May 20;9(5):e97175. doi: 10.1371/journal.pone.0097175 (PMC4028199; doi:10.1371/journal.pone.0097175)
Supplement: Figure S2 — Powder X-ray diffractogram of the residuals after thermal decomposition at 800°C in the TG. No diffraction peaks can be observed due to that the sample is amorphous after thermal decomposition. (PDF) [file pone.0097175.s002.pdf]

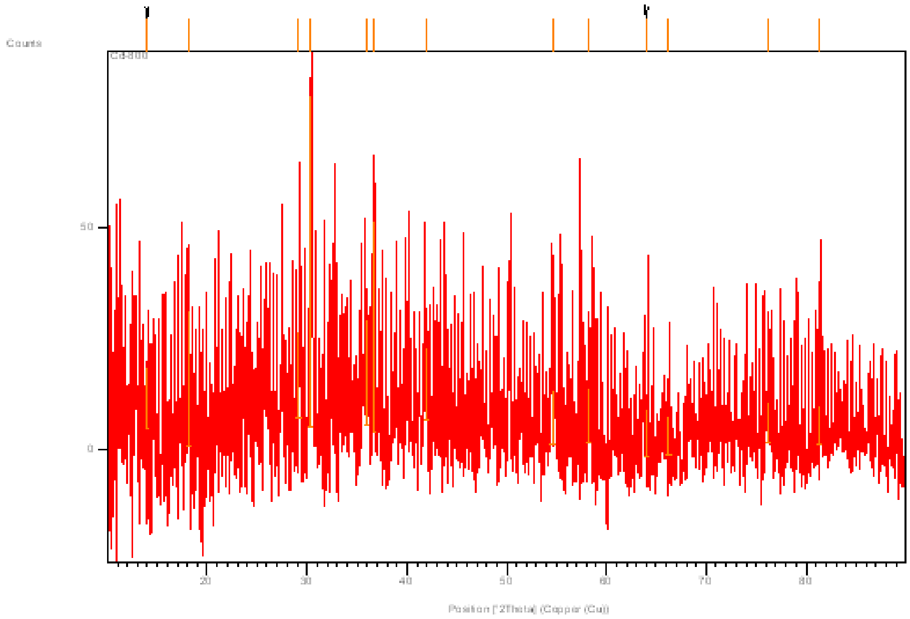

Figure S2 Powder X-ray diffractogram of the residuals after thermal decomposition at 800°C in the TG. No diffraction peaks can be observed due to that the sample is amorphous after thermal decomposition.
