# Supplementary material for: Synthesis, Crystal Structure and Thermal Decomposition of the New Cadmium Selenite Chloride, Cd4(SeO3)2OCl2
Source: PLoS One. 2014 May 20;9(5):e97175. doi: 10.1371/journal.pone.0097175 (PMC4028199; doi:10.1371/journal.pone.0097175)
Supplement: Figure S3 — Infrared Spectrum of sample residue after TGA. The sample was run on TGA instrument up to 800°C and residue was further characterized using Fourier Transform Infra-Red (FTIR) spectrophotometer {FTIR-4100, JASCO-CE, PerkinElmer, UK} to study the vibrational analysis. (PDF) [file pone.0097175.s003.pdf]

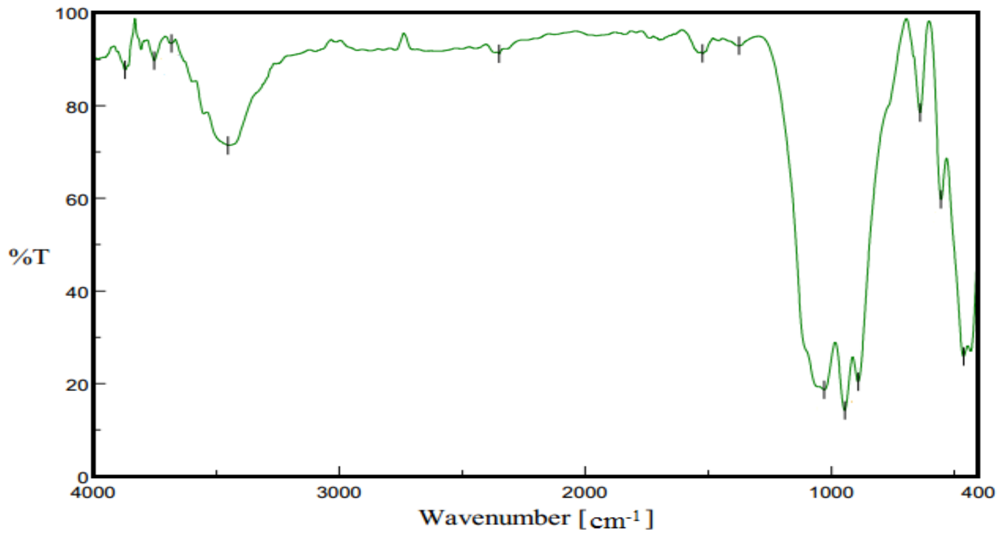

Figure S3 Infrared Spectrum of sample residue after TGA. The sample was run on TGA instrument upto 800°C and residue was further characterized using Fourier Transform Infra-Red (FTIR) spectrophotometer {FTIR-4100, JASCO-CE, PerkinElmer, UK} to study the vibrational analysis.

The infrared spectrum is plotted between transmittance intensity (%T) and wave number (cm^{-1}). The major peaks in this spectrum are located at 410, 462.832, 554.434, 639.287, 890.952, 945.913, 1028.84, 3453.88 cm^{-1} as shown the above figure. All the peaks are positioned lower than 1000 cm^{-1} and correlated to M-O vibrations [A] except 3453.88 cm^{-1} which is due to O-H stretching vibration as some water is absorbed during pellet formation. The peak at 639.287 cm^{-1} and 410 cm^{-1} are attributed to CdO [C]. The peak at 554.434 cm^{-1} also correlates with Cd-O vibration. [D].This indicates that residue left behind is CdO after sample decomposition and other peaks might be other phases of CdO.

References

[A] Rao, C.N.R., Chemical Applications of Infrared Spectroscopy, Academic Press, NewYork and London, 1963.

[B] (a) Gajbhiye N S, Ningthoujam R S, Ahmed A, Panda D K, Umre S S and Sharma S J, Proc of ASID, 06, 8-12, New Delhi, 2006, pp.5. (b) Amrut S Lanje, Raghumani S Ningthoujam, Satish J Sharma, Ramchandra B Pode, Luminescence and electrical resistivity properties of cadmium oxide nanoparticles, Indian journal of pure and applied physics, 49 (2011) 234-234.

[C] Mahesha N, Arunkumar Lagashetty, Synthesis And Characterisation Of Nanosized Cadmium Oxide, Vol. 2 (2013) 73-75.

[D] S. Sakthivel and D. Mangalaraj, Cadmium Oxide Nano Particles by Sol-Gel and VapourLiquid-Solid Methods, Nano Vision, Vol.1 (2011) 47-53.
