# Supplementary material for: Synthesis, Crystal Structure and Thermal Decomposition of the New Cadmium Selenite Chloride, Cd4(SeO3)2OCl2
Source: PLoS One. 2014 May 20;9(5):e97175. doi: 10.1371/journal.pone.0097175 (PMC4028199; doi:10.1371/journal.pone.0097175)
Supplement: Table S1 — Fractional atomic coordinates and isotropic or equivalent isotropic displacement parameters (Å2). (PDF) [file pone.0097175.s004.pdf]

Table S1 Fractional atomic coordinates and isotropic or equivalent isotropic displacement parameters (Å^2)

**Elements**	**Wyckoff**	**x**	**y**	**z**	**Uiso**	**Uiso*/Ueq**	**BVS**
Cd1	*8d*	0.2500	0.0000	0.2500	1.000	0.01109 (9)	2.052
Cd2	*8f*	0.2500	0.2500	0.2500	1.000	0.01314 (9)	1.960
Cd3	*16m*	0.5000	0.108696 (18)	0.107487 (17)	1.000	0.01667 (8)	1.900
Se1	*16m*	0.0000	0.15885 (2)	0.12291 (2)	1.000	0.01007 (9)	4.009
Cl1	*16o*	0.3367 (3)	0.19303 (13)	0.0000	0.490(3)	0.0189 (5)	0.718
Cl2	*16o*	0.2866 (3)	0.04827 (15)	0.0000	0.510(3)	0.0256 (6)	0.650
O3	*8i*	0.5000	0.0000	0.1832 (2)	1.000	0.0092 (7)	1.984
O4	*16m*	0.0000	0.26024 (17)	0.16461 (16)	1.000	0.0172 (6)	2.166
O5	*32p*	0.1809 (3)	0.12105 (12)	0.17187 (12)	1.000	0.0179 (4)	2.024
