# Supplementary material for: Synthesis, Crystal Structure and Thermal Decomposition of the New Cadmium Selenite Chloride, Cd4(SeO3)2OCl2
Source: PLoS One. 2014 May 20;9(5):e97175. doi: 10.1371/journal.pone.0097175 (PMC4028199; doi:10.1371/journal.pone.0097175)
Supplement: Table S2 — Selected bond distances and angles (Å,o) in Cd4(SeO3)2OCl2. (PDF) [file pone.0097175.s005.pdf]

Table S2 Selected bond distances and angles (Å,^{o}) in $Cd_4(SeO_3)_2OCl_2$

Cd1—O3^{i}	2.1822 (18)	Cd3—O5	2.614 (2)
Cd1—O3	2.1822 (19)	Cd3—O5^{viii}	2.614 (2)
Cd1—O5^{ii}	2.3787 (19)	Cd3—Cl2	2.6280 (16)
Cd1—O5^{iii}	2.3787 (19)	Cd3—Cl2^{viii}	2.6280 (16)
Cd1—O5^{i}	2.3787 (19)	Cd3—Cd3^{ix}	3.3682 (5)
Cd1—O5	2.3788 (19)	Se1—O5	1.690 (2)
Cd2—O4^{iv}	2.3791 (17)	Se1—O5^{x}	1.690 (2)
Cd2—O4^{v}	2.3791 (17)	Se1—O4	1.733 (3)
Cd2—O4^{vi}	2.3791 (17)	Cl1—Cl1^{xi}	2.179 (4)
Cd2—O4	2.3791 (17)	Cl1—Cl2	2.273 (3)
Cd2—O5^{vii}	2.476 (2)	Cl1—Cl1^{viii}	2.404 (4)
Cd2—O5	2.476 (2)	Cl1—Cd3^{xii}	2.5912 (15)
Cd2—O5^{vi}	2.476 (2)	Cl2—Cl2^{xiii}	1.496 (5)
Cd2—O5^{iii}	2.476 (2)	Cl2—Cd3^{xii}	2.6280 (16)
Cd3—O3	2.146 (2)	O3—Cd3^{ix}	2.146 (2)
Cd3—O4^{vi}	2.265 (3)	O3—Cd1^{ix}	2.1822 (19)
Cd3—Cl1^{viii}	2.5911 (15)	O4—Cd3^{vi}	2.265 (3)
Cd3—Cl1	2.5912 (15)	O4—Cd2^{v}	2.3791 (17)
O3^{i}—Cd1—O3	180.00 (15)	O3—Cd3—Cl2	99.52 (7)
O3^{i}—Cd1—O5^{ii}	97.45 (7)	O4^{vi}—Cd3—Cl2	129.61 (7)
O3—Cd1—O5^{ii}	82.55 (7)	Cl1^{viii}—Cd3—Cl2	86.20 (5)
O3^{i}—Cd1—O5^{iii}	82.55 (7)	Cl1—Cd3—Cl2	51.63 (7)
O3—Cd1—O5^{iii}	97.45 (7)	O5—Cd3—Cl2	78.44 (6)
O5^{ii}—Cd1—O5^{iii}	180.00 (8)	O5^{viii}—Cd3—Cl2	150.91 (6)
O3^{i}—Cd1—O5^{i}	82.55 (7)	O3—Cd3—Cl2^{viii}	99.52 (7)
O3—Cd1—O5^{i}	97.45 (7)	O4^{vi}—Cd3—Cl2^{viii}	129.61 (7)
O5^{ii}—Cd1—O5^{i}	75.92 (10)	Cl1^{viii}—Cd3—Cl2^{viii}	51.63 (7)
O5^{iii}—Cd1—O5^{i}	104.08 (10)	Cl1—Cd3—Cl2^{viii}	86.20 (5)
O3^{i}—Cd1—O5	97.45 (7)	O5—Cd3—Cl2^{viii}	150.91 (6)
O3—Cd1—O5	82.55 (7)	O5^{viii}—Cd3—Cl2^{viii}	78.44 (6)
O5^{ii}—Cd1—O5	104.08 (10)	Cl2—Cd3—Cl2^{viii}	73.41 (8)
O5^{iii}—Cd1—O5	75.92 (10)	O3—Cd3—Cd3^{ix}	38.29 (7)
O5^{i}—Cd1—O5	180.0	O4^{vi}—Cd3—Cd3^{ix}	153.71 (7)
O4^{iv}—Cd2—O4^{v}	101.86 (10)	Cl1^{viii}—Cd3—Cd3^{ix}	120.28 (4)
O4^{iv}—Cd2—O4^{vi}	78.66 (10)	Cl1—Cd3—Cd3^{ix}	120.28 (4)
O4^{v}—Cd2—O4^{vi}	172.35 (13)	O5—Cd3—Cd3^{ix}	94.20 (4)
O4^{iv}—Cd2—O4	172.35 (13)	O5^{viii}—Cd3—Cd3^{ix}	94.20 (4)
O4^{v}—Cd2—O4	78.66 (10)	Cl2—Cd3—Cd3^{ix}	69.13 (5)
O4^{vi}—Cd2—O4	101.86 (10)	Cl2^{viii}—Cd3—Cd3^{ix}	69.13 (5)
O4^{iv}—Cd2—O5^{vii}	75.87 (8)	O3—Cd3—O4^{vi}	115.42 (11)
O4^{v}—Cd2—O5^{vii}	62.98 (8)	O3—Cd3—Cl1^{viii}	147.90 (6)
O4^{vi}—Cd2—O5^{vii}	124.18 (8)	O4^{vi}—Cd3—Cl1^{viii}	82.56 (7)
O4—Cd2—O5^{vii}	97.84 (8)	O3—Cd3—Cl1	147.90 (6)
O4^{iv}—Cd2—O5	124.18 (8)	O4^{vi}—Cd3—Cl1	82.56 (7)
O4^{v}—Cd2—O5	97.85 (8)	Cl1^{viii}—Cd3—Cl1	55.27 (9)
O4^{vi}—Cd2—O5	75.87 (8)	O5^{vi}—Cd2—O5^{iii}	156.29 (9)
O4—Cd2—O5	62.98 (8)	O3—Cd3—O5	77.84 (5)
O5^{vii}—Cd2—O5	156.29 (9)	O4^{vi}—Cd3—O5	75.09 (5)
O4^{iv}—Cd2—O5^{vi}	97.84 (8)	Cl1^{viii}—Cd3—O5	134.02 (6)

$O4^{v}$—Cd2—$O5^{vi}$	124.18 (8)	Cl1—Cd3—O5	82.03 (6)
$O4^{vi}$—Cd2—$O5^{vi}$	62.98 (8)	O3—Cd3—$O5^{viii}$	77.84 (5)
O4—Cd2—$O5^{vi}$	75.87 (8)	$O4^{vi}$—Cd3—$O5^{viii}$	75.09 (5)
$O5^{vii}$—Cd2—$O5^{vi}$	72.43 (9)	$Cl1^{viii}$—Cd3—$O5^{viii}$	82.03 (6)
O5—Cd2—$O5^{vi}$	112.72 (9)	Cl1—Cd3—$O5^{viii}$	134.02 (6)
$O4^{iv}$—Cd2—$O5^{iii}$	62.98 (8)	O5—Cd3—$O5^{viii}$	127.96 (9)
$O4^{v}$—Cd2—$O5^{iii}$	75.87 (8)	Cl1—Cd3—O5	82.03 (6)
$O4^{vi}$—Cd2—$O5^{iii}$	97.84 (8)	O5—Se1—$O5^{x}$	104.00 (14)
O4—Cd2—$O5^{iii}$	124.18 (8)	O5—Se1—O4	95.69 (9)
$O5^{vii}$—Cd2—$O5^{iii}$	112.72 (9)	$O5^{x}$—Se1—O4	95.69 (9)
O5—Cd2—$O5^{iii}$	72.43 (9)		

Symmetry codes: (i) −*x*+1/2, −*y*, −*z*+1/2; (ii) *x*, −*y*, *z*; (iii) −*x*+1/2, *y*, −*z*+1/2; (iv) *x*+1/2, *y*, −*z*+1/2; (v) −*x*, −*y*+1/2, −*z*+1/2; (vi) −*x*+1/2, −*y*+1/2, *z*; (vii) *x*, −*y*+1/2, −*z*+1/2; (viii) −*x*+1, *y*, *z*; (ix) −*x*+1, −*y*, *z*; (x) −*x*, *y*, *z*; (xi) −*x*+1/2, −*y*+1/2, −*z*; (xii) *x*, *y*, −z; (xiii) *x*, −*y*, −*z*.
