# Supplementary material for: Synthesis, Crystal Structure and Thermal Decomposition of the New Cadmium Selenite Chloride, Cd4(SeO3)2OCl2
Source: PLoS One. 2014 May 20;9(5):e97175. doi: 10.1371/journal.pone.0097175 (PMC4028199; doi:10.1371/journal.pone.0097175)
Supplement: Table S3 — Results from Bond Valence Sum (BVS) calculations for Cd4(SeO3)2OCl2. (PDF) [file pone.0097175.s006.pdf]

Table S3 Results from Bond Valence Sum (BVS) calculations for $Cd_4(SeO_3)_2OCl_2$.

Se1

Se1-O4	1.235
Se1-O5×2	1.387
∑	4.0

Cd1

Cd1—O3×2	0.472
Cd1—O5×4	0.277
∑	2.1

Cd2

Cd2—O4×4	0.277
Cd2—O5×4	0.213
∑	2.0

Cd3

Cd3—O3	0.520
Cd3—O4	0.377
Cd3—O5×2	0.147
Cd3—Cl1*	0.359
Cd3—Cl2*	0.325
∑	1.9

*Two half occupied Cl1 and two half occupied Cl2 that together give one Cl1 and one Cl2.

O1

O3—Cd1×2	0.472
O3—Cd3 ×2	0.520
∑	2.0

O2

O4—Cd2×2	0.277
O4—Cd3	0.377
O4—Se1	1.235
∑	2.2

O3

O5—Se1	1.387
O5—Cd1	0.277
O5—Cd2	0.213
O5—Cd3	0.147
∑	2.0
